# The London Dermatological Society

**Published:** 1912-07-06

**Authors:** 


### The London Dermatological Society.

ITS OBJECTS AND FUTURE.

The London Dermatological Society held its first dinner on Monday, July 1, which was made the occasion for the presentation of the Chesterfield Medal. The guest of the evening was Lord Chesterfield (the Lord Steward). Dr. Morgan Dockrell presided, and among other gentlemen present were Sir Frederick Hodgson, Col. P. J. Freyer, Dr. Campbell Thompson, Dr. Bartholomew, Mr. McAdam Eccles, Dr. Helena Hall, Dr. Knowsley Sibley (Hon. Treasurer of the Society), and Dr. Griffith (Hon. Secretary). Mr. McAdam Eccles, in proposing the toast of the Society, said that everybody felt that there was ample room for a Society of this kind and that its labours would meet with success. Dr. Morgan Dockrell thought that the Society had a great future. It had been of great use to many members of the medical profession, and its objects were educational, consultative, and social. Lord Chesterfield then presented the medal to Dr. Bartholomew, who said that the help given by the St. John's Hospital for Diseases of the Skin was conducive to the success of the Society, and it was only through that help being extended to him that made him the proud possessor of the Chesterfield Medal.

